# Modifications of Polymers through the Addition of Ultraviolet Absorbers to Reduce the Aging Effect of Accelerated and Natural Irradiation

**DOI:** 10.3390/polym14010020

**Published:** 2021-12-22

**Authors:** Gamal A. El-Hiti, Dina S. Ahmed, Emad Yousif, Omar S. A. Al-Khazrajy, Mustafa Abdallh, Saud A. Alanazi

**Affiliations:** 1 Cornea Research Chair, Department of Optometry, College of Applied Medical Sciences, King Saud University, P.O. Box 10219, Riyadh 11433, Saudi Arabia; saaalanazi@ksu.edu.sa; 2 Department of Medical Instrumentation Engineering, Al-Mansour University College, Baghdad 64021, Iraq; dina.saadi@muc.edu.iq; 3 Department of Chemistry, College of Science, Al-Nahrain University, Baghdad 64021, Iraq; emad.yousif@nahrainuniv.edu.iq (E.Y.); mustafa.abdallh@nahrainuive.edu.iq (M.A.); 4 Department of Chemistry, College of Education for Pure Science (Ibn Al-Haytham), University of Baghdad, Baghdad 64021, Iraq; omar.s.a@ihcoedu.uobaghdad.edu.iq

**Keywords:** plastics, polyvinyl chloride, photostabilizers, plastic photodegradation and photooxidation, recycling of plastics, photoirradiation

## Abstract

The photooxidative degradation process of plastics caused by ultraviolet irradiation leads to bond breaking, crosslinking, the elimination of volatiles, formation of free radicals, and decreases in weight and molecular weight. Photodegradation deteriorates both the mechanical and physical properties of plastics and affects their predicted life use, in particular for applications in harsh environments. Plastics have many benefits, while on the other hand, they have numerous disadvantages, such as photodegradation and photooxidation in harsh environments and the release of toxic substances due to the leaching of some components, which have a negative effect on living organisms. Therefore, attention is paid to the design and use of safe, plastic, ultraviolet stabilizers that do not pose a danger to the environment if released. Plastic ultraviolet photostabilizers act as efficient light screeners (absorbers or pigments), excited-state deactivators (quenchers), hydroperoxide decomposers, and radical scavengers. Ultraviolet absorbers are cheap to produce, can be used in low concentrations, mix well with polymers to produce a homogenous matrix, and do not alter the color of polymers. Recently, polyphosphates, Schiff bases, and organometallic complexes were synthesized and used as potential ultraviolet absorbers for polymeric materials. They reduced the damage caused by accelerated and natural ultraviolet aging, which was confirmed by inspecting the surface morphology of irradiated polymeric films. For example, atomic force microscopy revealed that the roughness factor of polymers’ irradiated surfaces was improved significantly in the presence of ultraviolet absorbers. In addition, the investigation of the surface of irradiated polymers using scanning electron microscopy showed a high degree of homogeneity and the appearance of pores that were different in size and shape. The current work surveys for the first time the use of newly synthesized, ultraviolet absorbers as additives to enhance the photostability of polymeric materials and, in particular, polyvinyl chloride and polystyrene, based mainly on our own recent work in the field.

## 1. Introduction

Ultraviolet (UV) light has harmful effects on materials used in outdoor applications. Plastics suffer photooxidation when exposed to harsh conditions (high temperature, sunlight for long duration, and humidity) in the presence of oxygen. Plastic degradation, as a result of UV light absorption, leads to discoloration, cracks, and loss of mechanical and physical properties [1,2]. Photooxidation resembles autooxidation due to long-term heat aging, except that the driving force is UV light and not heat [3]. Therefore, during plastic manufacturing, measures should be taken to ensure that the materials will last longer and to inhibit photooxidation and photodegradation processes.

The polymerization technique was developed over the years to allow the production of plastics on an industrial scale. There has been a massive increase in the production of plastics in recent years [4]. The scale of polyvinyl chloride (PVC) production has increased over the years from 3 million tons in 1965 to over 40 million tons in 2018 and is expected to grow further to 60 million tons in 2025 [5]. PVC can be produced in different forms and shapes, using both suspension and emulsion polymerization [6]. Plastic waste is a challenge, and there is a need for not only effective recycling but cutting the waste at the source. Therefore, further developments in plastic are still needed to keep the environment clean and to elongate the lifetime of plastic [7,8].

Plastic contains polymeric chains that are based on carbon, hydrogen, and heteroatoms (e.g., sulfur, oxygen, or nitrogen). Polystyrene (PS), polypropylene (PP), polyethylene (PE), PVC, polyethylene terephthalate (PET), and polyurethane (PU) represent 75–80% of Europe’s plastic consumption (Table 1) [9,10]. These polymers have either C–C or C–heteroatom backbones, and their properties are highly dependent on the repeating units [11].

Plastics are highly involved in our daily lives, from household items to very complex medical equipment. They are used in construction materials (e.g., windows, panels, glazing, coatings, siding, roofing, flooring, fencing, and decoration), furniture, offices, agriculture (e.g., mulch film, materials for greenhouses, and production of sacks), transportation (e.g., bodywork and production of protective coatings), flame and smoke retardants (high content of chlorine; 57% by weight), insulators, and others [6]. Polycarbonate plastic has a low thermal conductivity (k) and, therefore, is better than conventional glazing agents [12]. The demand for plastic has extensively increased due to its unique mechanical and physical properties (e.g., light weight, strength, resistance to corrosion and chemicals) and low manufacturing cost. In addition, the shape and properties of plastics can be manipulated based on the application. However, UV radiation has a negative effect on plastic (e.g., rigid PVC) lifetime and leads to the loss of its strength. The solar irradiation of PVC causes discoloration and the emission of toxic volatiles, which hinders its use in outdoor applications [13]. PVC is still used as a construction material, but it can be replaced by polyolefins, which are less harmful but cost more.

The degradation of plastics is of major concern from an environmental perspective in terms of potential hazards to living organisms. The degradation of plastics takes place under either abiotic or biotic (e.g., biodegradation) conditions [14]. Biodegradation is highly dependent on environmental factors, which vary based on the type of polymer. Color changes and the crazing of plastic are early signs of degradation, followed by surface cracking and the formation of small fragments [15]. Floating plastics in seas and oceans are moderately affected by temperatures, solar radiation, and oxygen through photoinitiated oxidative degradation. For abiotic degradation, the contributing factors are sunlight and oxygen, and they affect the plastic through a hydrolysis process [16].

**Figure 1:**
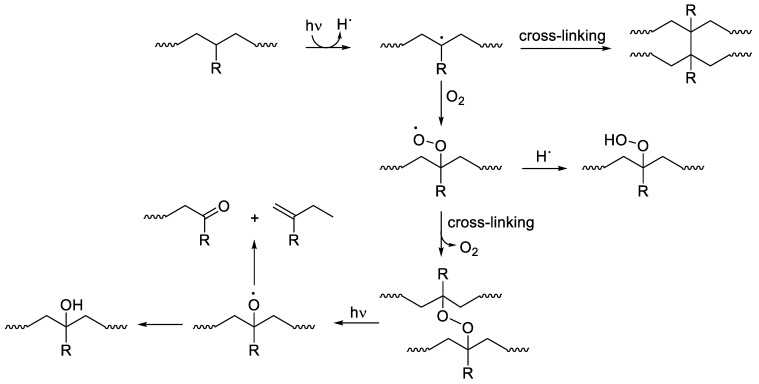
Abiotic degradation pathways for PE (R = H), PP (R = Me), and PS (R = Ph).

Three steps (initiation, propagation, and termination) are involved in plastic degradation [17]. The first step is initiated through solar or thermal initiators and leads to the formation of free radicals. Photoinitiation is not likely for both PE and PP, since they do not have unsaturated chromophores in their skeletons that are responsible for the absorption of light [18]. Impurities or abnormalities within plastics allow for the production of free radicals leading to C–H bonds cleavage in the backbone of polymers [19,20]. In the presence of oxygen, free radicals produce peroxy reactive moieties in the propagation step. In addition, hydroperoxides can be produced, leading to the autoxidation of polymers [21]. In the propagation step, crosslinking or chain scission takes place [22]. The deactivation of free radicals occurs in the termination step, leading to stable products. In the presence of oxygen, the formation of oxygen-containing moieties is expected, which leads to a photoinitiated degradation process. The chain scission and crosslinking (termination) of oxygenated species leads to the formation of olefins (unsaturated polymeric chains), aldehydes, and ketones (Figure 1) [23].

Plastic natural degradation is initiated through photodegradation followed by thermo-oxidative degradation [24]. Sun UV light provides the energy needed to initiate the incorporation of oxygen into the polymeric chains [25]. Plastics are degraded to small polymeric fragments, and then metabolized by microorganisms in the surrounding environment. Microorganisms tend to convent the polymeric chain carbons to either carbon dioxide or biomolecules [26,27]. However, such a process is very slow (taking up to 50 years) for the complete degradation of plastics [28]. Chromophores present within the skeleton of polymers absorb visible or UV light, and therefore initiate the photodegradation process [29,30]. Photodegradation takes place either in the presence of oxygen (e.g., photooxidation) or in its absence (e.g., chain crosslinking or bond breaking). When polymers (e.g., polyolefins) are exposed to heat, UV light, or mechanical stress in the presence of oxygen, they produce free radicals that initiate the oxidation process. Therefore, plastics should be stabilized to inhibit the oxidative processes to increase the half-life time of materials [31].

Plastic weathering involves changes in the physical, mechanical, and chemical properties of polymers, particularly at the surface [32]. Solar energy, moisture (e.g., rain, snow, or humidity), oxidants (e.g., ozone or atomic or singlet oxygen), and air pollutants (e.g., sulfur dioxide, nitrogen oxides, or polycyclic hydrocarbons) are responsible for these changes [33]. Uneven discoloration, surface cracks, or loss of strength are the most common changes within plastics due to degradation [34]. Climate change and the rise in global temperatures accelerate polymers’ weathering, and impurities (e.g., traces of metals or oxidants) present in additives increase the rate of photodegradation [35].

**Figure 2:**
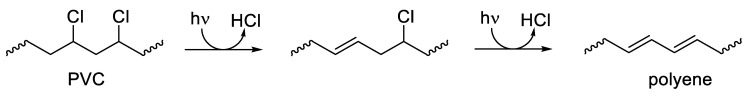
Dechlorination of PVC and formation of polyene polymeric chains.

PVC is a synthetic plastic that is similar to PP, but the backbone carbons are attached to chlorine atoms instead of hydrogens. PVC is one of the most common manufactured plastics [36]. Due to the high content of chlorine, PVC is hard and stiff. In addition, PVC is polar due to the presence of C–Cl bonds and is soluble in many solvents, particularly those containing polar atoms such as ethers (e.g., dioxane, tetrahydrofuran, ketones, or nitrobenzene). It has a low cost, is durable, has excellent performance, is easily molded, and can be obtained in different shapes that are suitable for many applications. PVC is commonly used in packaging, health care devices, toys, construction materials, electrical wire insulation, clothes, and furnishing [5,6]. For outdoor applications, PVC photostability should be enhanced through the addition of suitable additives to inhibit its photodegradation. The dechlorination of PVC is autocatalytic, which leads to the formation of –C=C–. The formation of unsaturated double bonds within the backbone of PVC leads to its photodegradation, in which small fragments and polyene residues are produced (Figure 2) [37].

Plastic recycling has received attention recently due to the large volume of waste that it generates [38]. Pyrolysis and incineration of PVC are not recommended due to the high level of hydrogen chloride (HCl) and other toxic volatiles produced [39]. The most common methods for PVC recycling include chemical and mechanical techniques. Mechanical recycling is preferred when the PVC waste composition is known [40]. On the other hand, the chemical recycling of PVC converts plastics back to chemicals that can be reused in the polymerization process. The development of techniques and instrumentation for the separation of PVC from the waste stream is still important to allow for the recovery of most wasted PVC.

Recently, our research was directed towards investigating the use of newly synthesized aromatic compounds and those that include organometallics as potential UV absorbers. We made some progress in this field, which is reported in the current work.

## 2. Photostabilization of Polymers

The photostabilization of polymers has received much attention recently, in order to find efficient methods to inhibit their photochemical degradation. Additives are added to polymers to improve their performance and mechanical and thermal properties [41]. The additives act as stabilizers, fillers, plasticizers, softeners, lubricants, colorants, flame retardants, blowing agents, crosslinking agents, and UV absorbers. UV stabilizers are capable of reducing the rate of photooxidation of polymeric materials [42]. Various parameters such as color, stability, compatibility, volatility, and cost should be taken into consideration in the selection of additives. The additives should be capable of absorbing the harmful UV radiation and dissipating the energy as heat over time at a harmless rate to the polymers. Some polymers, such as polymethyl methacrylate and polytetrafluoroethylene are highly stable and do not require the addition of photostabilizers for outdoor applications. Moderately photostable polymers, such as polyvinyl fluoride and polyvinylidene fluoride have a lifetime of a few years in outdoor applications and can be used without the addition of photostabilizers. On the other hand, poorly stable polymers such as PVC, PS, and polyamides have a short lifetime (less than a year), and therefore require the use of UV stabilizers for outdoor use [43,44]. The polymer additives act as UV screeners, excited state deactivators, hydroperoxide decomposers, and radical scavengers [45].

In the case of PVC, the dipoles along the polymer chain, due to the presence of chlorine atoms, lead to a high level of secondary valency forces, and therefore reduce chain flexibility. The van der Waals force within PVC chains is insignificant in cohesion due to the relative bulkiness of the chlorine atoms. The polarized groups within plasticizers bound to polymer dipoles and the non-polar moiety act as shields between polymer dipoles. Therefore, a reduction in dipole bonding between polymer chains, less overall cohesion, and an increase in the flexibility of movement are observed [46]. The incorporation of a low concentration of plasticizers can lead to flexible products but increases the stiffness at the same time. The addition of plasticizers in a low concentration leads to an increase in the crystallinity level of the polymers [47]. Therefore, it appears that plasticized PVC has a degree of microcrystalline structure. PVC shows solvated regions, which are flexible due to the presence of a plasticizer and non-solvated crystalline areas. The PVC crystallite network structure has an impact on the toughness and strength and is responsible for the variation of PVC properties [48,49].

## 3. Photostabilization of Polymers Using UV Absorbers

**Figure 3:**
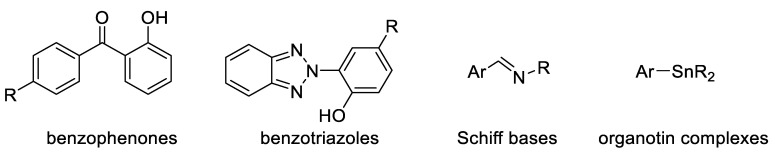
Structures of some common UV absorbers.

UV absorbers play an important role in absorbing harmful radiation from light and dissipating it as harmless thermal energy [50,51,52]. In addition, they block the formation of free radicals that are produced at the early stages of degradation. The most common industrial UV absorbers are titanium oxide, carbon black, benzophenones, and triazoles (e.g., hydroxylbenzophenone and hydroxyphenylbenzotriazole), while the most common additives used recently for research include Schiff bases and organometallic complexes (Figure 3).

**Figure 4:**
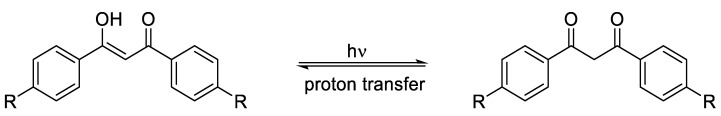
Hydroxybenzophenones energy dissipation through a proton transfer.

These additives have unique UV absorbance characteristics. For example, benzophenone-containing additives absorb UV strongly in the short-wavelength region through a proton transfer or tautomeric structure equilibrium (Figure 4). They are more efficient compared with the additives containing benzotriazole. Benzophenone-based UV absorbers have unique properties such as a low cost, low toxicity, and good resistance to water and acids [53].

**Figure 5:**
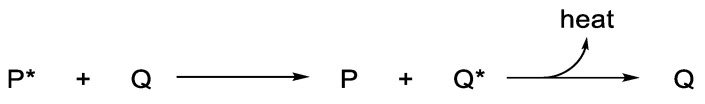
UV absorbers act as quenchers for the excited state energy of polymers. * Represents the excited state.

Triazoles have high molar extinction coefficients (5 × 10^6^ cm^−1^M^−1^) and absorb the most destructive wavelength of light (280–370 nm), which is highly involved in polymer degradation. The excitation of benzotriazoles takes place once the UV light is absorbed; the benzotriazoles then dissipate the energy through either heat release, involving a hydrogen transfer, or fluorescence emission [54]. In addition, UV absorbers act as quenchers (Q) for the triplet excited state of the polymer chromophoric group (P *), followed by the release of energy as harmless heat (Figure 5) [55]. Similarly, metal complexes act as effective UV quenchers due to their low excitation coefficients and quench the triplet state of the carbonyl groups in polyolefins [56,57,58].

Recently, we synthesized a range of UV stabilizers (e.g., aromatics, heterocycles, Schiff bases, organometallic complexes, and polyphosphates) and tested their efficiency for the stabilization of polymers [59,60,61,62,63,64,65,66,67,68,69,70,71,72,73,74,75,76,77,78,79,80,81,82,83,84,85,86,87,88,89,90,91,92,93,94]. These additives, at a low concentration of 0.5% by weight, led to a significant improvement in the photostability of polymers. The stabilization effect that the UV absorbers induced in polymers was examined using infrared spectroscopy, the determination of weight and molecular weight, and inspection of the surface of polymers.

## 4. Morphological Study of the Surface of Irradiated Polymers in the Presence of Additives

**Figure 6:**
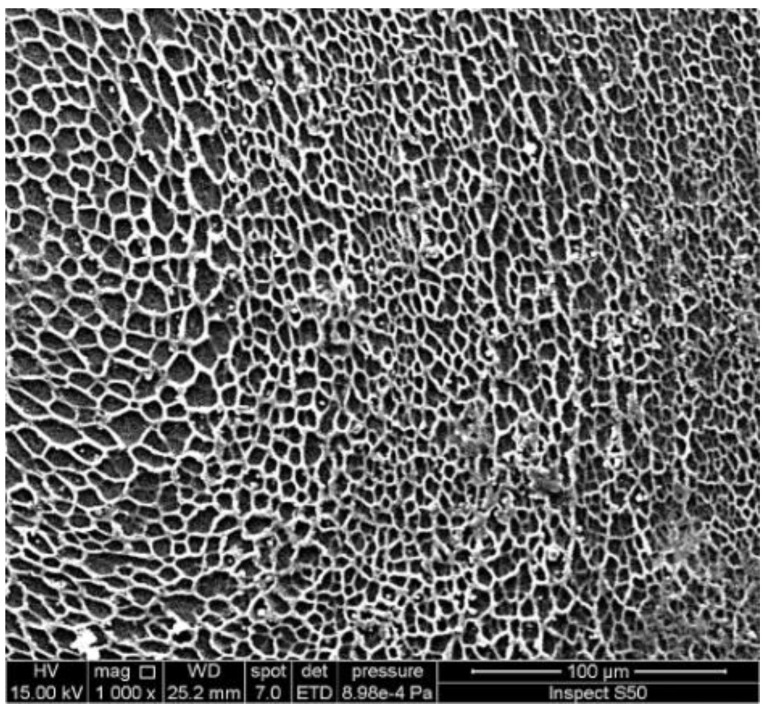
SEM image of the surface of an irradiated PVC film blended with a polyphosphate containing a benzidine moiety.

**Figure 7:**
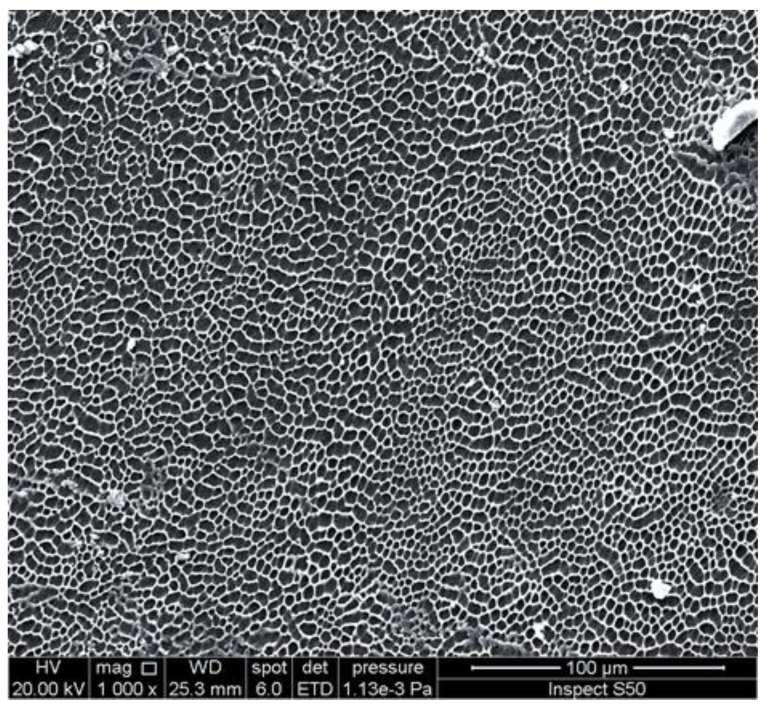
SEM image of the surface of an irradiated PVC film blended with a 4-methoxybenzoic acid-Sn complex.

An investigation of the surface morphology of polymers can provide important information about the damage that takes place due to weathering and the changes in particles’ size and shape. Scanning electron microscopy (SEM) and field-emission scanning electron microscopy (FESEM) are used to provide information about distortion, variation on the surface, the shape and size of particles, and homogeneity [95,96,97,98,99]. The irradiated polymers show the presence of cracks, holes, lumps, spots, and amorphous and irregular surfaces. These changes are mainly due to dehydrochlorination, chain scission, and crosslinking. However, the damage on the surface of polymers was limited in the presence of UV absorbers compared with the blank polymers. In some cases, the irradiated films containing additives showed the interesting changes that took place on the surface [100,101,102,103,104]. For example, the SEM image of the surface of the irradiated PVC film blended with a polyphosphate containing benzidine at 25 °C, showing the formation of hexagonal pores (Figure 6) with a honeycomb-like structure, which do not appear within the blank irradiated material [74]. Increasing the irradiation time by up to 300 h led to an increase in the number of hexagonal pores. The reasons for the formation of such a structure are not clear, but it could be a result of the elimination of HCl at a low rate and its scavenging by the Sn complex. Crosslinked materials could produce honeycomb-like structures as a result of water stabilization [105,106,107,108,109,110]. For example, the irradiation of a thin film of crosslinked polystyrene, at 25 °C for 6 h, produced a honeycomb-like structure [106]. The irradiation of polyacrylic glycidyl ether for a short duration led to the formation of a honeycomb film [107]. Similarly, the SEM image of the irradiated PVC film containing a 4-methoxybenzoic acid-Sn complex showed a honeycomb-like structure (Figure 7) [95].

**Figure 8:**
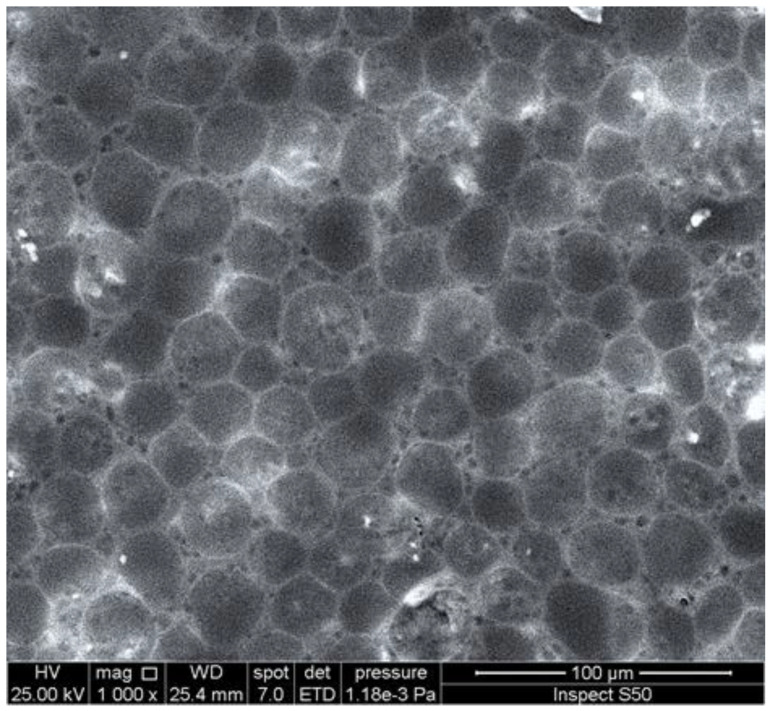
SEM image of the surface of an irradiated PVC film blended with a Schiff base containing a thiadiazole moiety in the presence of nickel chloride.

The irradiated PVC film, blended with a Schiff base and containing a thiadiazole moiety in the presence of nickel chloride, showed the presence of hexagonal pores on the surface (Figure 8) [72]. The presence of nickel ions is necessary to produce the honeycomb-like structure and to enhance the photostability of the polymeric materials [111]. The structure of the irradiated film was highly porous with a large surface area, possibly due to the incorporation of nickel ions within the polymer. The formation of a honeycomb structure depends on the type of solvent used during the fabrication process of the film, the length of the side-chain within the polymer, and the concentration of the polymer [112].

**Figure 9:**
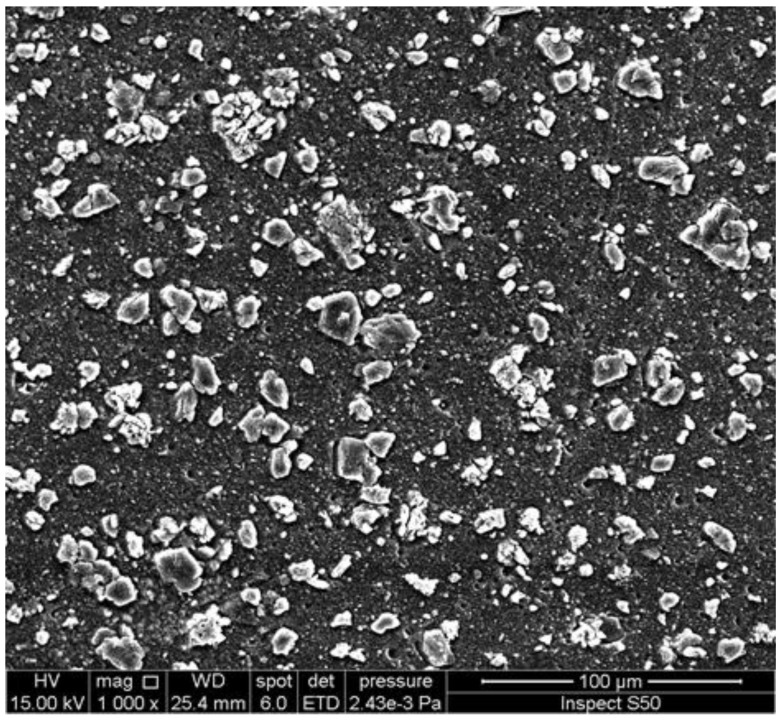
SEM image of the surface of an irradiated PVC film blended with a Schiff base of melamine.

**Figure 10:**
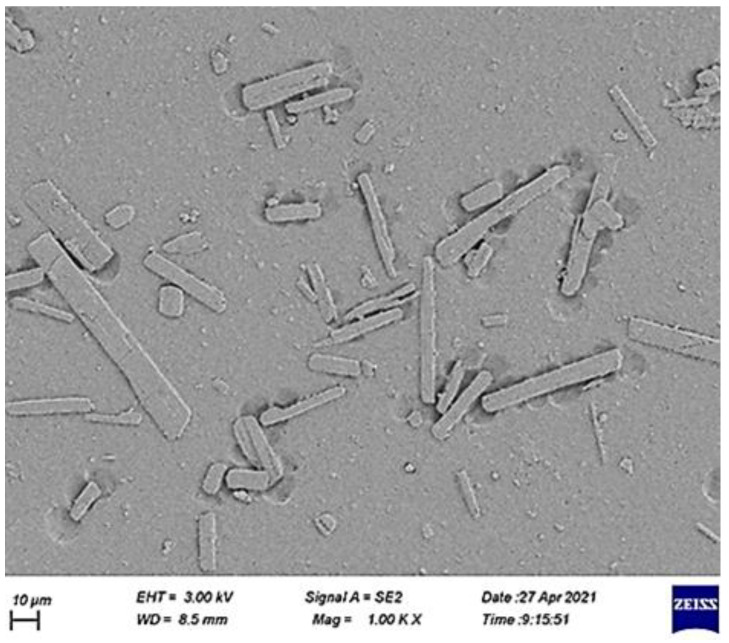
FESEM image of the surface of an irradiated PVC film blended with a trimethoprim-Sn complex.

The SEM image of the surface of an irradiated PVC film, blended with a melamine-Schiff base (Figure 9), showed ice-cube-like particles [75]. Meanwhile, the FESEM image of the surface of an irradiated PVC film, blended with a trimethoprim-Sn complex, showed rod-like particles (Figure 10) [93]. It is believed that the crosslinking and elimination of volatiles and hydrogen chloride at a slow rate are the reasons for the formation of the particles that have such shapes [113,114].

**Figure 11:**
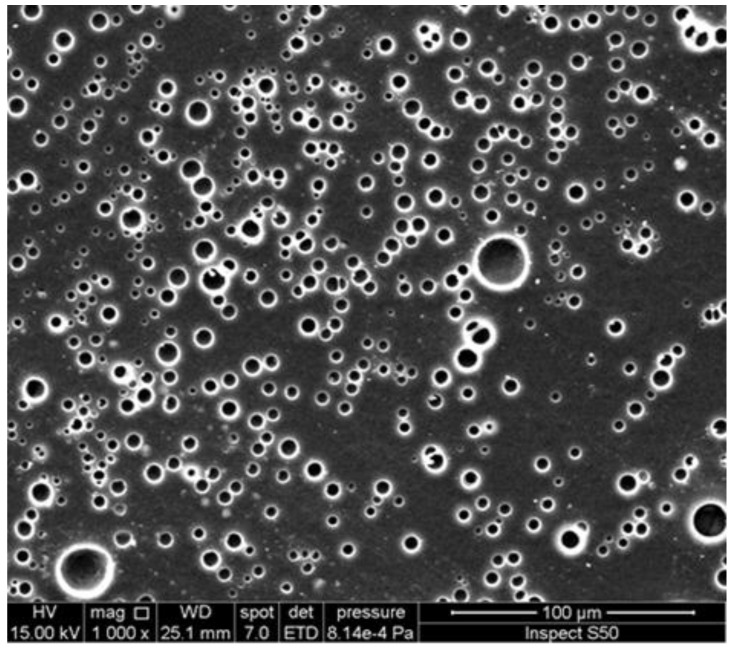
SEM image of the surface of an irradiated PS film blended with a Schiff base of biphenyl-3,3′,4,4′-tetraamine.

The PS film blended with a Schiff base of biphenyl-3,3′,4,4′-tetraamine showed spherical and embedded ellipsoid pores that have a diameter from 3.4 to 4.3 µm (Figure 11) after irradiation [73]. The formation of ball-like pores may be a result of the effective light absorption and porous structure of UV absorbers.

**Figure 12:**
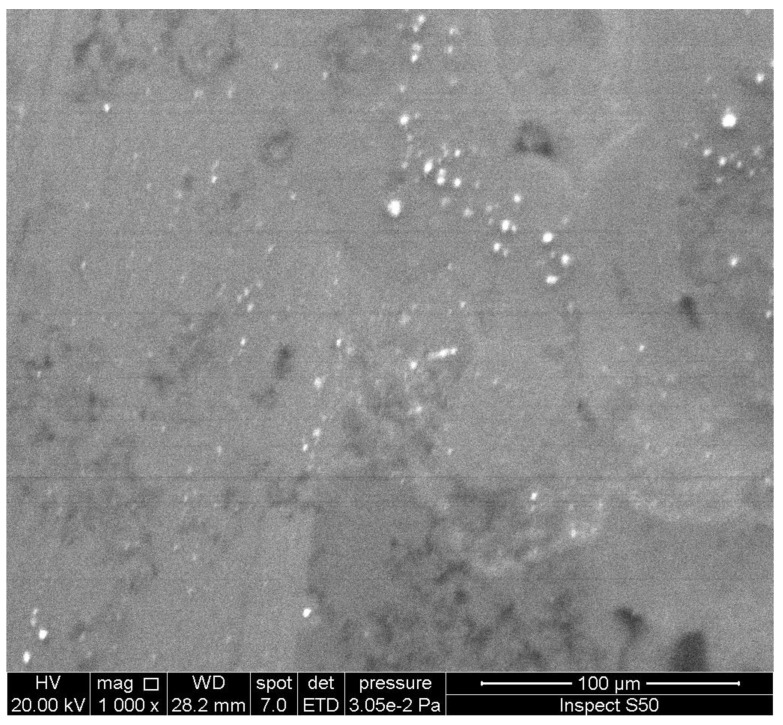
SEM image of the surface of an irradiated PVC film in the absence of any additive.

**Figure 13:**
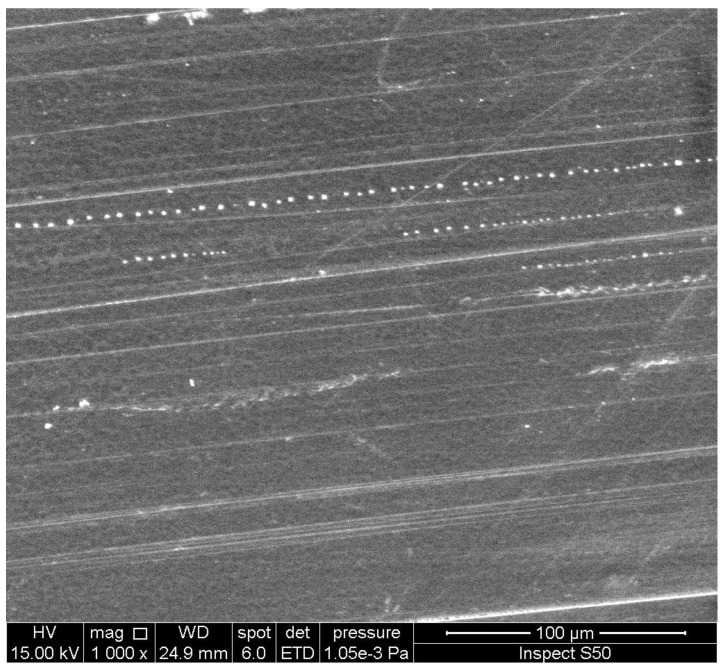
SEM image of the surface of an irradiated PS film in the absence of any additive.

For comparison, Figure 12 and Figure 13 show the SEM images of the blank PVC and PS films, respectively, in the absence of any additives after irradiation.

Atomic force microscopy (AFM) was used as a tool to measure the effectiveness of UV absorbers towards the stabilization of polymers [115,116,117]. The roughness factor (*Rq*) for the surface of the blank, irradiated polymers was always high compared with those obtained for the films blended with additives. Such an observation is evidence for the important role played by additives in stabilizing polymers upon irradiation. Highly aromatic (due to the resonance effect) UV additives that contain heteroatoms (due to coordination with the polymeric chain of PVC, for example) showed the most desirable stabilizing effect (Table 2).

## 5. Conclusions and Future Perspectives

Polymer stabilization is one of the most important processes that is used to elongate the lifetimes of plastic products. Plastics used in outdoor applications suffer in harsh environments and quickly lose their mechanical and physical properties. The proper solution for inhibiting the photooxidation of plastics due to the inevitable exposure to light and oxygen is through the addition of efficient ultraviolet absorbers that are capable of acting as efficient scavengers for light and blocking the formation of free radicals within the polymeric chains. The additives should absorb irradiation light directly and decompose peroxide species. In addition, they should be very compatible with the polymers, not alter the color, be used at a very low concentration, and be safe for the environment if released. Progress was made with the design and use of safe additives to enhance plastic stability and, in particular, polystyrene and polyvinyl chloride. Polyphosphates, Schiff bases, and organometallic complexes containing aromatic moieties showed the potential to be used as ultraviolet absorbers for plastics. The damage on the surface of irradiated plastics in the presence of ultraviolet absorbers is low compared with the blank films.

Since the additives are not linked to plastic through covalent bonds, they can be leached to the surrounding environments. The leakage of these chemicals followed by their degradation poses a danger to both animals and humans. Therefore, future research should be attention to the design, synthesis, and use of safe, non-toxic, and highly stable polymeric additives to suppress the degradation of plastic. Some progress was made, but there is still room for further improvements and modifications.

## Figures and Tables

**Table 1:** The most common plastics and their European demand [10].

Plastic (Repeating Unit)		Name	European Demand (%)
C–C Backbone	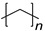	PE	29.6
	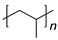	PP	19.9
	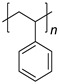	PS	7.1
	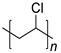	PVC	10.4
Heteroatoms in backbone	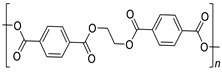	PET	6.9
	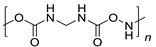	PU	7.4

**Table 2:** Reduction in the roughness factor *Rq* (by fold) of polymers in the presence of UV absorbers.

Polymer	UV Absorber	Organic Moiety	*Rq*	Reference
PS	Schiff base	Cephalexin	27.1	[92]
PS	Schiff base	Biphenyl-3,3′,4,4′-tetraamine	8.3	[73]
PS	Schiff base	1,2,3,4-Triazole-3-thiol	3.3	[64]
PVC	Polyphosphates	Benzidine	16.8	[68]
PVC	Schiff base	Biphenyl-3,3′,4,4′-tetraamine	3.6	[66]
PVC	Schiff base	Melamine	6.0	[75]
PVC	Ni complex	2-(4-Isobutylphenyl) propanoate	6.3	[65]
PVC	Sn complex	4-Methoxybenzoic acid	21.2	[94]
PVC	Sn complex	4-(Benzylideneamino) benzenesulfonamide	18.4	[91]
PVC	Sn complex	Ciprofloxacin	16.6	[70]
PVC	Sn complex	Trimethoprim	11.3	[93]
PVC	Sn complex	Telmisartan	9.4	[78]
PVC	Sn complex	Valsartan	7.4	[81]
PVC	Sn complex	Furosemide	6.6	[63]
PVC	Sn complex	Carvedilol	6.4	[88]
PVC	Sn complex	Naproxen	5.2	[77]

## Data Availability

Data are contained within the article.
